# Acute Kidney Injury in a Young Woman after Dilation and Curettage

**DOI:** 10.34067/KID.0000000000000519

**Published:** 2024-09-26

**Authors:** Sandipan Shringi, Boris Sinayuk, Eric Kerns

**Affiliations:** 1 Division of Kidney Disease and Hypertension, Rhode Island Hospital, Providence, Rhode Island; 2 Warren Alpert Medical School of Brown University, Providence, Rhode Island; 3 Department of Radiology, Rhode Island Hospital, Providence, Rhode Island

**Keywords:** AKI, renal hemodynamics, renal ischemia, women's health, imaging

## Abstract

**Figure:**
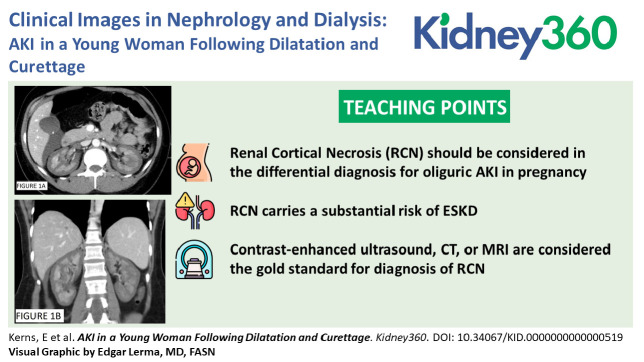

A 31-year-old woman with no medical history presented with hemorrhagic shock after dilation and curettage at 17 weeks gestation due to intrauterine fetal demise. Laboratory test results were consistent with disseminated intravascular coagulation. Serum creatinine was 2.0 mg/dl from a previous baseline of 0.6 mg/dl. She was transferred to the medical intensive care unit and treated with blood transfusions, fresh frozen plasma, and cryoprecipitate along with oxytocin, misoprostol, and thromboxane. She underwent uterine artery embolization because of a large intrauterine hematoma. During the next 6 days, serum creatinine increased to 13 mg/dl accompanied by anuria. A contrast-enhanced computed tomography (CT) scan of the abdomen and pelvis revealed bilateral reverse nephrostograms (reverse rim signs), with the renal cortex appearing hypodense to the renal medullae. There was a nonobstructing inferior vena cava thrombus and a right gonadal vein thrombus. Renal cortical necrosis (RCN) was diagnosed. She started hemodialysis for uremic symptoms and remained dialysis dependent for approximately 2 months. Six months after the diagnosis, the serum creatinine was 2.4 mg/dl with a creatinine clearance on 24-hour urine collection of 33 ml/min and subnephrotic proteinuria, 300 mg/d. The patient remains on low-dose loop diuretics and anticoagulation.

RCN is a severe and often irreversible form of ischemic AKI. It results from a prolonged reduction in renal arterial perfusion and is often accompanied by disseminated intravascular coagulation. Clinically, RCN presents as abrupt onset of oliguria or anuria, sometimes with gross hematuria and flank pain. The diagnosis is confirmed by contrast-enhanced ultrasound, CT, or MRI, which shows the absence of contrast in the renal cortex and collecting system with preserved enhancement of the medulla.^1^ The so-called reverse rim sign implies deranged cortical blood flow, mostly affecting the renal tubules and sparing the medulla and pyramids (Figure 1, A and B). These imaging findings correlate with necrosis of glomeruli and tubules on biopsy^2^ and autopsy.^1^ RCN on imaging can appear patchy when it is incomplete or diffuse when it is complete. This may provide prognostic information.^2^

**Figure 1:**
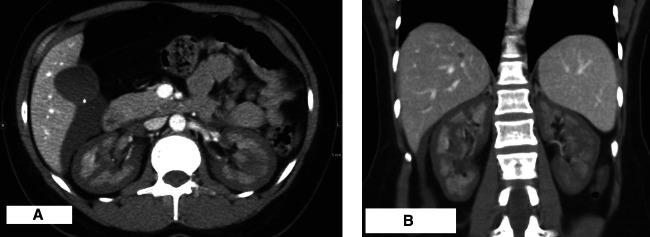
**Reverse rim sign in renal cortical necrosis.** (A) Axial contrast-enhanced CT image demonstrates symmetric cortical hypoenhancement with preserved medullary enhancement of the reverse rim sign. There is a small amount of fluid in the hepatorenal recess. (B) Coronal contrast-enhanced CT image demonstrates a similar finding of symmetric cortical renal hypoenhancement with preserved medullary enhancement of the reverse rim sign. CT, computed tomography.

The largest study of outcomes in RCN after obstetric catastrophes found that 100% of patients required dialysis at diagnosis and 40% remained dialysis dependent after 6 months.^3^ It is generally accepted that 30%–50% of patients with RCN will progress to ESKD. Other potential etiologies for RCN, apart from obstetric catastrophes, include sepsis, burns, trauma, pancreatitis, severe dehydration, and hemolytic uremic syndrome.

The pathophysiology of RCN is not known; however, a differential blood supply to the renal cortex compared with medulla and an absence of collaterals to cortex could explain the findings. Eighty-six percent of renal blood flow is directed to the cortex^4^ with separate extrarenal blood supply to the peripheral cortical rim and juxtamedullary portion.^5^ A fall in oxygen tension during states of hemorrhagic hypotension is, therefore, more severe in cortical tissue compared with medullary tissue, making it more susceptible to ischemic injury.

## Teaching Points

- RCN should be considered in the differential diagnosis for oliguric AKI in pregnancy.
- RCN carries a substantial risk of ESKD.
- Contrast-enhanced ultrasound, CT, or MRI is considered the gold standard for diagnosis of RCN.
